# Accelerometer-based physical activity levels among Mexican adults and their relation with sociodemographic characteristics and BMI: a cross-sectional study

**DOI:** 10.1186/s12966-015-0243-z

**Published:** 2015-06-20

**Authors:** Deborah Salvo, Catalina Torres, Umberto Villa, Juan A. Rivera, Olga L. Sarmiento, Rodrigo S. Reis, Michael Pratt

**Affiliations:** Michael and Susan Dell Center for Healthy Living, The University of Texas Health Science Center at Houston, School of Public Health (Austin regional campus), Austin, TX USA; Center for Nutrition and Health Research, National Institute of Public Health of Mexico, Cuernavaca, Morelos Mexico; Institute for Computational Engineering and Sciences, The University of Texas at Austin, Austin, TX USA; Schools of Medicine and Government, Universidad de los Andes, Bogotá, Colombia; Research Group of Physical Activity and Quality of Life (GPAQ), School of Health and Biosciences, Pontificia Universidade Católica do Paraná, Curitiba, Brazil; Department of Physical Education, Universidade Federal do Paraná, Curitiba, Brazil; Nutrition and Health Sciences Program, Hubert Department of Global Health, Rollins School of Public Health, Emory University, Atlanta, GA USA

**Keywords:** Physical activity, Accelerometry, Latin America, Correlates of activity, Bouts of activity, Epidemiology

## Abstract

**Background:**

The objectives of this study were to describe the accelerometer based total and bout-specific PA levels for a representative sample of adults from Cuernavaca, Mexico, and to examine the relationships with sociodemographic characteristics and BMI status.

**Methods:**

Cross sectional study of adults from Cuernavaca, Mexico (2011, *n* = 677). Participants wore Actigraph GT3X accelerometers for seven days and sociodemographic data was collected through a survey. Weight and height were objectively measured. Total minutes/week of moderate-to-vigorous PA (MVPA) and of MVPA occurring within bouts of at least ten minutes were obtained. Intensity-specific (moderate and vigorous) total PA and bouted-PA was also obtained. The relation of each PA variable with sex, age, socioeconomic status, education, marital status and BMI status was assessed using unadjusted and adjusted linear models.

**Results:**

The mean total MVPA among adults from Cuernavaca was 221.3 ± 10.0 (median = 178.3 min/week). Average MVPA within bouts was 65.8 ± 4.7 min/week (median = 30.0 min/week). 9.7 % of total MVPA occurred within bouts. Significant associations were found for total and bout-specific MVPA with being male (positive) and owning a motor vehicle (negative). Additional associations were found for intensity-specific PA outcomes. Mexican adults were more active during weekdays than weekends, suggesting that PA may be more strongly driven by necessity (transport) than by choice (leisure).

**Conclusions:**

This is the first study to objectively measure PA for a representative sample of Mexican adults in an urban setting. The sociodemographic correlates vary from those known from high income countries, stressing the need for more correlate studies from lower-to-middle income countries.

**Electronic supplementary material:**

The online version of this article (doi:10.1186/s12966-015-0243-z) contains supplementary material, which is available to authorized users.

## Background

Physical inactivity has been defined as a pandemic [1]. It is a risk factor for obesity, cardiovascular disease, type II diabetes, osteoporosis and many types of cancer [2, 3]. During 2008, 5.3 million deaths were attributable to physical inactivity worldwide [2]. In Mexico it is estimated that physical inactivity accounted for 4.4 % of total deaths and 1.2 % of total DALYS in 2004, making it a leading contributor to the burden of disease [4]. Currently, 71.2 % of Mexican adults are either overweight or obese [5], and the first two causes of death are cardiovascular diseases and type II diabetes [6]. The World Health Organization (WHO) recommends that adults engage in at least 150 min of moderate to vigorous physical activity (PA) per week, or 75 min of vigorous activity per week, to be done within bouts of at least ten minutes of sustained duration [7].

In many high-income countries (HIC), surveillance of population PA levels has been taking place for decades, and the characterization of sociodemographic factors associated with inactivity has been extensively documented [8]. This has not been the case for Mexico, where the study of physical activity as it relates to public health remains a nascent field [9]. PA measurement was included in the National Health and Nutrition Survey (ENSANUT, conducted every six years) in 2006, for a subsample of adults and adolescents through self-reported measures [10]. For the latest ENSANUT (2012), the short version of the International Physical Activity Questionnaire was administered to the entire sample, and this survey shows that 17.2 % of Mexican adults are inactive [5].

As in most countries, representative population-level PA data for Mexican adults is entirely based on self-report [5]. There are a number of concerns related to relying solely on self-report to estimate PA levels in populations. One of the main issues is the overestimation of time spent in moderate-to-vigorous PA [11]. The importance of using objective measures to accurately report PA levels for populations is now well recognized [8, 12]. Accelerometers are the most widespread research tools for measuring PA objectively, allowing the precise recording of time spent in PA by intensity level [8]. Nationally representative accelerometry data is only available for Canada, Norway, Portugal, Sweden and the US [8, 13]. Researchers have also used this tool to study levels of PA within bouts of at least ten minutes of continuous activity in different populations, since prolonged periods of MVPA yield important cardiovascular benefits [7, 14, 15]. Neither total nor bout-specific accelerometer-based PA levels have been reported for a representative sample of Mexican adults, and the sociodemographic correlates of accelerometer-based PA remain unknown for Mexicans. The identification of sociodemographic factors associated with inactivity is key to identify which population subgroups should be targeted by interventions, programs and policies for increasing PA in Mexico.

The purpose of this study was to describe the total and bout-specific levels of objectively measured PA among a representative sample of Mexican adults from the city of Cuernavaca. This study also identified sociodemographic characteristics related to total and bout-specific accelerometer-based PA among adults from Cuernavaca, Mexico.

## Methods

Cuernavaca is a mid-sized city in central Mexico (population: 365,168) [16]. Mean income per capita is 18,370 USD and Cuernavaca has a Human Development Index (HDI) of 0.86 (National HDI = 0.77) [17].

### Study design and sampling

This was a cross-sectional study, and was part of the IPEN-Mexico study (IPEN: International Physical Activity Environment Network) which has been described in detail elsewhere [9, 18, 19].

Data collection took place from April to September, 2011. A representative stratified multistage clustered sample was selected. Census tracts were the primary sampling units. All census tracts within the Municipality of Cuernavaca (*N* = 123) were stratified by high (above the median) and low (below the median) walkability. The walkability index was calculated using z-scores of intersection density (number of 4-way intersections over total area per census tract), land use mix (diversity of land use types per census tract, using a normalized entropy score ranging from 0 to 1) [20], proportion of commercial land use (over total census tract area) and net residential density (total residences over area destined for residential use per census tract) [18, 20]. Census tracts were also stratified by socioeconomic status (SES) based on quartiles (SES levels 1 to 4, based on average income using census information) [21]. The sample had eight strata, derived from the combination of walkability (high and low) and SES (1 to 4). Four census tracts were randomly selected per stratum, for a total of 32 census tracts in the study. Seven blocks were randomly selected per census tract (secondary sampling units). Finally, two to four households were randomly selected per block (tertiary sampling unit). One participant per household was selected for the study. In case of refusal, non-eligibility or not finding anyone at home after two visits, the household to the right (clockwise) was selected. Eligible participants were residents between 20 to 65 years, with no temporary or permanent disability precluding walking, who had been living at that address for at least six months. Further information on the sampling strategy has been reported elsewhere [19].

### Instruments

#### Accelerometers

PA was assessed with Actigraph GT3X accelerometers using sixty-second epochs and a sampling rate of 30 Hz. Participants were instructed to wear the accelerometer for seven days during waking hours, only removing it for water-based activities (e.g., showering, swimming).

#### Survey on sociodemographic characteristics

An interviewer-administered survey included items on sex, age, time of residence in the household, marital status, education, motor vehicle ownership, household characteristics and assets.

#### Scales

Tanita® scales with centigram precision were used to measure weight using standardized procedures [22].

#### Stadiometers

Fixed wooden stadiometers with milimetric precision were used to measure height using standardized procedures [22].

### Recruitment and data collection

Recruitment and data collection were done in person via home visits with a team of trained field workers. The first home visit was to inform the household that it had been randomly selected for the study. The aims and procedures of the study were explained and an eligible participant living in the household was invited to participate. Written informed consent was obtained for all participants, and an accelerometer was provided with instructions and a log, and an appointment was set for a second visit. Two monitoring phone calls during the week verified correct use of the accelerometer. During the second visit the survey was administered, weight and height were measured and accelerometer wear-time was verified. If wear-time verification during the second visit revealed that the minimum wear-time criteria had not been met, the participant was asked to re-wear the accelerometer for the required additional days, and a third home visit was scheduled to recover the device.

The study was approved by the Institutional Review Boards of Emory University and the National Institute of Public Health of Mexico.

### Accelerometer data verification and scoring

Data verification was done on site using Actilife 4.0 to download the data and MeterPlus 4.2 to verify wear time. A minimum of five days of at least ten valid hours per day was required. Periods of time of sixty or more consecutive zeros were considered as being indicative of non-wear time. Freedson cut-points for adults [23] were used to score the data using MeterPlus 4.2 in compliance with the IPEN protocol [24].

### Variables

A detailed description of all dependent and independent variables included in our analysis is found in Table 1. The following outcome variables were used: Total minutes of moderate PA per week (TMPA), total minutes of vigorous PA per week (TVPA), total minutes of moderate-to-vigorous PA per week (TMVPA), minutes of moderate PA per week within bouts (BMPA), minutes of vigorous PA per week (BVPA) and minutes of moderate-to-vigorous PA per week within bouts. Similarly, intensity-specific variables were generated to estimate total and bouted-PA for weekdays (Monday through Friday) and weekends. MVPA bouts were defined as having a minimum duration of ten minutes, with at least 80 % of the bout corresponding to MVPA. Therefore, break periods of lower PA intensities within a bout were allowed, but could only constitute up to 20 % of the bout. This was done to account for real-life situations (e.g., someone taking a brisk walk in their neighborhood that had to stop at a stop-light in an intersection before proceeding). Each single break within a given bout was allowed a maximum duration of 2 min (e.g., if a bout lasted 20 min, up to 4 min could correspond to total break-time, but each single break within the bout could only last a maximum of 2 min).

**Table 1:** Socio-Demographic characteristics and BMI status of adults from Cuernavaca, Mexico, 2011

Variable	Number	Weighted %^a^
Total	677.0	100.0
Male	302.0	48.0
Age		
*<=35 years*	222.0	33.4
*35 < years < =50*	263.0	39.0
*50 < years < =65*	192.0	27.6
SES^b^		
*Low*	201.0	31.2
*Medium*	165.0	24.0
*Medium-High*	198.0	28.9
*High*	113.0	15.9
Education		
*Some Elementary*	36.0	5.0
*Complete Elementary*	67.0	10.1
*Some Middle School*	23.0	3.9
*Complete Middle School*	140.0	21.1
*Some High School*	29.0	4.4
*Complete Highschool*	162.0	23.2
*Some College*	34.0	5.5
*Complete College or more*	186.0	26.8
Motor vehicle ownership		
*Car*	363.0	53.2
*Motorcycle*	32.0	5.1
*Either*	371.0	54.7
Marital status		
*Single*	166.0	25.0
*Married or living with someone*	438.0	65.3
*Separated or Divorced*	56.0	7.4
*Widower*	17.0	2.4
BMI Status		
*Under-nutrition (BMI < 20)*	22.0	3.2
*Normal (20 < =BMI < 25)*	165.0	24.2
*Overweight (25 < =BMI < 30)*	278.0	40.9
*Obese (BMI > =30)*	212.0	31.7

^a^Weighted for probability of selection and non-response by sex

^b^SES: Classifications based on quartiles of SES-index. SES-index based on household characteristics and assets

Independent variables included: sex, age, individual-level SES, education, motor vehicle ownership, marital status and BMI. Individual level SES was obtained using a centered z-scored index based on twenty-five items on household characteristics and assets used in the 2006 Mexican National Health and Nutrition Survey to estimate individual-level SES among Mexicans [10].

### Statistical analysis

#### Descriptive analysis

Prevalence of having at least 150 min per week of TMVPA and BMVPA were obtained, as well as mean minutes per week of MVPA, MPA and VPA (for total and bouted PA). Since weekly minutes of PA were not normally distributed, values for the 25th, 50th and 70th percentile were also obtained. Results were stratified by sex, age, and SES. Results were weighted by total accelerometer wear time, probability of selection, and non-response by sex.

#### Regression analysis

Unadjusted and adjusted linear regression models were run to study the association between sociodemographic variables and each PA outcome. The adjusted models included all the studied sociodemographic variables and also controlled for total wear-time. Significance was considered when p ≤ 0.05.

#### Analytical software

MeterPlus 4.2 was used to generate all total-PA variables. The code to generate all bout-specific PA variables was conceived and developed by UV using MatLab 7.7 (The MathWorks Inc., Natick, MA, USA). Statistical analyses were performed using SAS 9.3 (SAS Institute Inc., Cary, NC, USA). The *surveymeans* and *surveyfreq* procedures in SAS were used for the descriptive analyses. The *surveyreg* procedure of SAS was employed for the regression analyses. By obtaining design-based estimates and using the Taylor series linearization method, [25] SAS’s *surveyreg* allows for the linear modeling of non-normal and non-symmetric outcomes [26], while accounting for the complex stratified multistage clustered study design [27].

## Results

The response rate, based on eligible adults who agreed to participate in the study, was 58.9 %. Table 1 shows the sociodemographic characteristics of the final study sample (*n* = 677). The mean age was 42.0 years, 48.0 % were male, 32.3 % had education beyond high school, 54.7 % owned at least one motor vehicle (car or motorcycle), 40.9 % were overweight and 31.7 % were obese. Eight participants were excluded due to missing valid accelerometry data (i.e., did not meet minimum wear-time criteria), 2 due to missing BMI, and 15 due to missing sociodemographic variables, leaving a total analytic sample of 652. No significant differences were found between the full study sample and the analytic sample.

The mean and median TMVPA among adults from Cuernavaca were 221.3 ± 10.0 and 178.3 mins/wk, respectively. Average BMVPA was 65.8 ± 4.7 mins/wk (median = 30.0 mins/wk). 9.7 % of TMVPA occurred within bouts as defined for this study. Males had a higher average TMVPA and BMVPA than females (270.1 ± 13.9 vs. 175.2 ± 7.5 mins/wk, and 82.2 ± 7.6 vs. 50.3 ± 5.1 mins/wk), and spent a higher proportion of their TMVPA and BMVPA in VPA than females (4.0 vs. 1.6 %, and 9.0 vs. 4.3 %). 58.6 % of Mexican adults accumulated 150 or more minutes per week of TMVPA, and 13.9 % did so for BMVPA (Table 2).

**Table 2:** Prevalence of achieving 150 min per week or more of moderate-to-vigorous physical activity^a^, by sociodemographic characteristics and BMI status, among adults from Cuernavaca, Mexico, 2011

	Total (unbouted) MVPA	MVPA within bouts of at least 10 minutes^b^
	≥150 min per week	≥150 min per week
	% (95 % confidence interval)	% (95 % confidence interval)
Overall	58.6 (54.0, 63.3)	13.9 (10.8, 17.0)
Sex		
Male	67.9 (61.5, 74.4)	19.5 (15.1, 23.9)
Female	49.8 (43.4, 56.2)	8.6 (4.5, 12.8)
Age		
≤ 35 years	68.7 (61.2, 76.2)	16.2 (10.5, 22.0)
35 < years ≤ 50	58.8 (51.9, 65.6)	11.9 (7.9, 15.9)
50 < years ≤ 65	46.4 (39.0, 53.9)	13.9 (9.3, 18.6)
SES^c^		
Low (Q1)	68.1 (58.6, 77.6)	16.3 (10.9, 21.6)
Medium (Q2)	60.6 (52.0, 69.1)	16.5 (8.6, 24.5)
Medium-High (Q3)	52.6 (44.2, 60.9)	10.9 (7.9, 13.9)
High (Q4)	48.3 (38.5, 58.0)	10.8 (3.1, 18.5)
Education		
< High School	62.1 (55.5, 68.7)	12.9 (8.3, 17.5)
High School	60.9 (54.0, 67.8)	14.5 (8.1, 20.9)
> High School	52.3 (45.0, 59.6)	14.9 (10.3, 19.5)
Motor Vehicle Ownership		
No	68.9 (62.3, 75.6)	20.8 (14.7, 26.9)
Yes	50.1 (44.7, 55.6)	8.2 (5.8, 10.6)
Marital Status		
Single	63.7 (53.4, 74.0)	19.7 (11.5, 27.9)
Married^d^	58.4 (52.2, 64.5)	13.1 (9.2, 16.9)
Divorced^e^	47.6 (32.8, 62.3)	4.9 (0.0, 13.0)
BMI Status		
BMI <25	56.8 (48.4, 65.2)	19.0 (12.5, 25.5)
25 ≤ BMI <30	60.4 (54.6, 66.2)	12.0 (7.8, 16.3)
BMI ≥ 30	57.9 (50.3, 65.5)	12.0 (6.8, 17.1)

Prevalences are weighted for probability of selection and non-response by sex

^a^Cut-point (≥150 min per week) was selected to enhance comparability with reports from other countries/settings using this cut point for their accelerometer-based results; yet, these prevalences should not be interpreted as representing the proportion of the population meeting WHO recommendations for physical activity, which are based on studies relying on self-reported physical activity

^b^MVPA-bouts are defined as having at least 10 min in duration, with ≥80 % corresponding to MVPA, and with each break below the threshold for MVPA (<1952 counts per minute) lasting 2 min maximum

^c^Based on quartiles of individual SES index, constructed using centralized z-scores from a set of questions on household characteristics and assets per participant. The index excluded motor vehicle ownership and education

^d^Includes “living with someone”

^e^Includes “divorced” and “widower”

On average, Mexican adults spent 169.7 ± 7.7 (median = 138.7) minutes during weekdays engaging in MVPA, versus 52.6 ± 2.8 (median = 35.5) minutes during weekends (Table 3). The daily average MVPA was also higher for weekdays (52.1 ± 3.8, median = 22.1) than weekends (14.3 ± 1.2, median = 0.0). The lower amount of PA during weekends is consistent for both sexes and PA intensities (Table 3).

**Table 3:** Physical activity during weekdays and weekends, by sex, among Mexican adults from Cuernavaca, Mexico, 2011

Outcome	*Weekdays*				*Weekends*			
	Mean (SE)	Q1	Med	Q3	Mean (SE)	Q1	Med	Q3
*Overall (n = 630)*								
Total Activity								
Mon-Fri: MVPA total mins	169.7 (7.7)	66.1	138.7	237.3	52.6 (2.8)	15.6	35.5	71.3
Average daily MVPA mins	33.9 (1.5)	13.2	27.7	47.5	26.3 (1.4)	7.8	17.8	35.6
Mon-Fri: MPA total minutes	163.9 (7.3)	64.9	135.0	229.4	51.6 (2.8)	15.6	35.4	69.4
Average daily MPA mins	32.8 (1.5)	13.0	27.0	45.9	25.8 (1.4)	7.8	17.7	34.7
Mon-Fri: VPA total mins	5.7 (0.8)	0.0	0.0	1.9	1.0 (0.2)	0.0	0.0	0.0
Average daily VPA mins	1.1 (0.2)	0.0	0.0	0.4	0.5 (0.1)	0.0	0.0	0.0
Activity within bouts^a^								
Mon-Fri: MVPA bouted mins	52.1 (3.8)	0.0	22.1	68.5	14.3 (1.2)	0.0	0.0	17.7
Average daily bouted-MVPA mins	10.4 (0.8)	0.0	4.4	13.7	7.1 (0.6)	0.0	0.0	8.8
Mon-Fri: MPA bouted mins	48.0 (3.6)	0.0	21.1	65.0	13.6 (1.2)	0.0	0.0	16.5
Average daily bouted-MPA mins	9.6 (0.7)	0.0	4.2	13.0	6.8 (0.6)	0.0	0.0	8.3
Mon-Fri: VPA bouted mins	4.1 (0.6)	0.0	0.0	0.0	0.7 (0.2)	0.0	0.0	0.0
Average daily bouted-VPA mins	0.8 (0.1)	0.0	0.0	0.0	0.3 (0.1)	0.0	0.0	0.0
*Male (n = 279)*								
Total Activity								
Mon-Fri: MVPA total mins	205.7 (10.5)	96.3	157.3	279.8	67.4 (4.7)	20.6	52.9	95.7
Average daily MVPA mins	41.1 (2.1)	18.7	31.5	56.0	33.7 (2.3)	10.3	26.5	47.8
Mon-Fri: MPA total minutes	196.3 (9.9)	89.8	149.0	270.6	65.8 (4.6)	19.9	52.8	89.7
Average daily MPA mins	39.3 (2.0)	18	29.8	54.1	32.9 (2.3)	9.9	26.4	44.8
Mon-Fri: VPA total mins	9.4 (1.4)	0.0	0.0	5.9	1.6 (0.3)	0.0	0.0	0.6
Average daily VPA mins	1.9 (0.3)	0.0	0.0	1.2	0.8 (0.1)	0.0	0.0	0.3
Activity within bouts^a^								
Mon-Fri: MVPA bouted mins	65.0 (6.3)	0.0	31.9	85.1	19.1 (2.1)	0.0	0.0	27.2
Average daily bouted-MVPA mins	13.0 (1.3)	0.0	6.4	17.0	9.6 (1.0)	0.0	0.0	13.6
Mon-Fri: MPA bouted mins	58.5 (6.1)	0.0	30.5	73.5	18.1 (2.1)	0.0	0.0	24.2
Average daily bouted-MPA mins	11.7 (1.2)	0.0	6.1	14.7	9.0 (1.0)	0.0	0.0	12.1
Mon-Fri: VPA bouted mins	6.5 (1.1)	0.0	0.0	1.7	1.1 (0.2)	0.0	0.0	0.0
Average daily bouted-VPA mins	1.3 (0.2)	0.0	0.0	0.3	0.5 (0.1)	0.0	0.0	0.0
*Female (n = 351)*								
Total Activity								
Mon-Fri: MVPA total mins	136.8 (6.5)	53.5	110.7	190.1	39.2 (2.1)	11.7	28.2	55.8
Average daily MVPA mins	27.4 (1.3)	10.7	22.1	38.0	19.6 (1.0)	5.8	14.1	27.9
Mon-Fri: MPA total minutes	134.5 (6.3)	52.7	110.7	189.0	38.7 (2.1)	11.7	27.9	55.3
Average daily MPA mins	26.9 (1.3)	10.5	22.1	37.8	19.4 (1.0)	5.8	14.0	27.7
Mon-Fri: VPA total mins	2.3 (0.5)	0.0	0.0	0.0	0.4 (0.2)	0.0	0.0	0.0
Average daily VPA mins	0.5 (0.1)	0.0	0.0	0.0	0.2 (0.1)	0.0	0.0	0.0
Activity within bouts^a^								
Mon-Fri: MVPA bouted mins	40.2 (4.3)	0.0	13.6	57.3	9.8 (1.5)	0.0	0.0	10.3
Average daily bouted-MVPA mins	8.0 (0.9)	0.0	2.7	11.5	4.9 (0.8)	0.0	0.0	5.1
Mon-Fri: MPA bouted mins	38.4 (4.2)	0.0	13.6	55.1	9.5 (1.5)	0.0	0.0	10.3
Average daily bouted-MPA mins	7.7 (0.8)	0.0	2.7	11.0	4.7 (0.7)	0.0	0.0	5.1
Mon-Fri: VPA bouted mins	1.9 (0.5)	0.0	0.0	0.0	0.3 (0.2)	0.0	0.0	0.0
Average daily bouted-VPA mins	0.4 (0.1)	0.0	0.0	0.0	0.2 (0.1)	0.0	0.0	0.0

Mon-Fri PA outcome variables: total valid weekday minutes registered for given PA outcome*5/total valid weekdays; and weighed for total valid weekday wear time

Sat-Sun PA outcome variables: total valid weekend minutes registered for given PA outcome*2/total valid weekend days; and weighed for total valid weekend wear time

^a^Only activity registered within MVPA-bouts of at least 10 min duration, with ≥80 % corresponding to MVPA is reported

After adjusting for all covariates, being male and owning a motor vehicle were significantly associated with PA among Mexican adults (Table 4). Males had 109.9 ± 13.2 (p < 0.0001) more mins/wk of TMVPA, and 37.0 ± 8.0 (p < 0.0001) more mins/wk of BMVPA than females. Owning a motor vehicle (> = 1) was associated with having 83.7 ± 17.2 mins/wk (p < 0.0001) less of TMVPA, and 50.6 ± 10.2 mins/wk (p < 0.0001) less BMVPA than non-motor vehicle owners.

**Table 4:** Associations of total and intensity-specific objectively measured minutes per week of MVPA with sociodemographic variables among adults, Cuernavaca, Mexico, 2011

Sociodemographic Characteristics and BMI	Minutes per week of total (not bouted) PA			Minutes per week of PA within bouts^a^ of at least 10 min		
	MVPA	MPA	VPA	MVPA	MPA	VPA
	Regression estimate ± SE	Regression estimate ± SE	Regression estimate ± SE	Regression estimate ± SE	Regression estimate ± SE	Regression estimate ± SE
	(*p* value)	(*p* value)	(*p* value)	(*p* value)	(*p* value)	(*p* value)
Sex						
Female	*Reference*	*Reference*	*Reference*	*Reference*	*Reference*	*Reference*
Male	109.9 ± 13.20	101.9 ± 12.9	8.0 ± 1.5	37.0 ± 8.0	32.0 ± 7.9	5.0 ± 1.2
	(<0.0001)	(<0.0001)	(<0.0001)	(<0.0001)	(0.0004)	(0.0003)
Age						
≤35 years	*Reference*	*Reference*	*Reference*	*Reference*	*Reference*	*Reference*
35 < years ≤ 50	−15.9 ± 16.2	−10.9 ± 15.6	−5.0 ± 2.7	−1.0 ± 8.0	1.7 ± 7.5	−2.6 ± 1.9
	(0.336)	(0.490)	(0.073)	(0.906)	(0.825)	(0.178)
50 < years ≤ 65	−48.1 ± 13.9	−42.8 ± 13.6	−5.3 ± 2.8	10.1 ± 7.1	12.2 ± 7.0	−2.1 ± 2.2
	(0.002)	(0.004)	(0.067)	(0.165)	(0.094)	(0.331)
SES^b^						
Low (Q1)	*Reference*	*Reference*	*Reference*	*Reference*	*Reference*	*Reference*
Medium (Q2)	−21.6 ± 24.7	−19.7 ± 23.9	−1.9 ± 3.2	−2.0 ± 10.1	0.0 ± 9.8	−2.1 ± 2.4
	(0.391)	(0.418)	(0.564)	(0.841)	(0.997)	(0.397)
Medium-High (Q3)	−13.4 ± 24.6	−9.9 ± 24.4	−3.5 ± 3.2	4.1 ± 11.3	7.4 ± 11.4	−3.4 ± 3.2
	(0.592)	(0.689)	(0.288)	(0.722)	(0.519)	(0.296)
High (Q4)	−21.0 ± 28.6	−17.5 ± 28.5	−3.5 ± 4.0	9.8 ± 15.6	12.8 ± 15.8	−3.1 ± 3.6
	(0.469)	(0.544)	(0.380)	(0.536)	(0.425)	(0.399)
Education						
< High School	*Reference*	*Reference*	*Reference*	*Reference*	*Reference*	*Reference*
High School	−5.3 ± 21.8	−7.0 ± 21.2	1.7 ± 1.6	−3.7 ± 9.4	−4.8 ± 9.0	1.1 ± 1.2
	(0.808)	(0.744)	(0.298)	(0.698)	(0.597)	(0.350)
> High School	−25.4 ± 23.0	−32.9 ± 22.6	7.5 ± 3.7	2.6 ± 12.3	−3.5 ± 11.3	6.1 ± 2.9
	(0.281)	(0.158)	(0.049)	(0.836)	(0.760)	(0.046)
Motor vehichle ownership						
No	*Reference*	*Reference*	*Reference*	*Reference*	*Reference*	*Reference*
Yes	−83.7 ± 17.2	−79.9 ± 16.7	−3.7 ± 2.8	−50.6 ± 10.2	−48.9 ± 9.9	−1.7 ± 2.2
	(<0.0001)	(<0.0001)	(0.196)	(<0.0001)	(<0.0001)	(0.436)
Marital status						
Single	*Reference*	*Reference*	*Reference*	*Reference*	*Reference*	*Reference*
Married^c^	18.6 ± 16.3	16.5 ± 14.8	2.1 ± 2.9	6.2 ± 9.7	4.8 ± 8.6	1.4 ± 2.1
	(0.264)	(0.275)	(0.477)	(0.533)	(0.582)	(0.520)
Divorced^d^	−40.4 ± 25.5	−39.1 ± 23.8	−1.2 ± 3.2	−29.2 ± 18.3	−28.1 ± 16.0	−1.1 ± 2.6
	(0.125)	(0.112)	(0.700)	(0.122)	(0.091)	(0.666)
BMI Status						
BMI < 25	*Reference*	*Reference*	*Reference*	*Reference*	*Reference*	*Reference*
25 ≤ BMI < 30	−19.1 ± 17.9	−15.2 ± 17.1	−3.9 ± 2.7	−17.8 ± 10.8	−14.2 ± 10.2	−3.5 ± 2.3
	(0.297)	(0.382)	(0.155)	(0.113)	(0.176)	(0.134)
BMI > 30	−31.5 ± 19.8	−27.6 ± 19.1	−3.9 ± 2.7	−16.9 ± 11.6	−13.5 ± 10.8	−3.5 ± 2.5
	(0.125)	(0.160)	(0.155)	0.156)	(0.223)	(0.184)

All models are adjusted to account for the multistage clustered design of the study, for total accelerometer wear-time, and for all sociodemographic variables and BMI

^a^MVPA-bouts are defined as having at least 10 min in duration, with ≥80 % corresponding to MVPA, and with each break below the threshold for MVPA (<1952 counts per minute) lasting 2 min maximum

^b^Based on quartiles of individual SES index, constructed using centralized z-scores from a set of questions on household characteristics and assets per participant. The index excluded motor vehicle ownership and education

^c^Also includes “living with someone”

^d^Also includes “divorced” and “widower”

Although BMI status (being overweight or obese, in comparison to normal weight) was not significantly associated with TMVPA or BMVPA, we did find a significant linear relationship (*p* = 0.049) between BMI score (continuous) and TMVPA (-3.20 ± 1.56) after adjusting for covariates.

The intensity-specific regression analysis (Table 4) showed significant positive associations between TMPA (101.9 ± 12.9, *p* < 0.0001), TVPA (8.0 ± 1.5, *p* < 0.0001), BMPA (32.0 ± 7.9, *p* = <0.0004) and BVPA (5.0 ± 1.2, *p* = 0.0003) with being male after adjusting for all covariates. Being 51 to 65 years was negatively related to TMPA (-42.8 ± 13.6, *p* = 0.004) and had marginal significance for TVPA (-5.3 ± 2.8, *p* = 0.067), but no significant association was found for either BMPA or BVPA. Having an education level higher than high school was positively associated with both TVPA (7.5 ± 3.7, *p* = 0.049) and BVPA (6.1 ± 2.9, *p* = 0.046), but not to TMPA or BMPA. Finally, motor vehicle ownership was only significantly negatively associated with the moderate PA outcomes (TMPA:-79.9 ± 16.7, *p* < 0.0001; and BMPA:-48.9 ± 9.9, *p* < 0.0001), but not with TVPA or BVPA.

The studied individual level variables (sociodemographic characteristics and BMI) explained 19 % of the variability of TMVPA and 10 % of the variability of BMVPA (based on R-squared values). The studied variables contribute more highly to explaining the variability of MPA than of VPA (R-squared values: TMPA = 0.18, BMPA = 0.10, TVPA = 0.09, BVPA = 0.06).

## Discussion

This is the first study to report objectively measured levels of PA for a representative sample of Mexican adults from an urban setting (Cuernavaca, Morelos). MVPA was significantly related to BMI score among Mexican adults. Higher levels of MVPA occur during weekdays for Mexicans, and being female and owning a motor vehicle (independent of SES and other sociodemographic factors) were strongly inversely related to MVPA (total and within bouts).

The percentage of the population with at least 150 min per week of MVPA, within bouts of at least 10 min (13.9 %) was found to be considerably lower than those reported by the latest ENSANUT based on self-report (82.6 % of Mexican adults reported meeting guidelines) [5]. Our results are consistent with studies from HIC showing that when considering only activity within bouts, and using objective measures instead of self-report, the proportion of adults achieving at least 150 min per week within bouts of MVPA is very low [11, 28, 29]. As Troiano et al. have stressed, the WHO recommendation for adults of 150 min per week of MVPA within bouts of at least 10 min is based on epidemiologic studies which relied on self-reported PA (capturing behaviors), and therefore findings from studies with accelerometer-derived PA (capturing movement), like ours, should not be interpreted as representing the proportion of the population meeting the WHO recommendations for PA [30]. Up to date, PA recommendations based on objectively measured PA are not available, but these will likely require a significantly lower volume of MVPA per week than current “self-report-based” guidelines [30]. Nonetheless, the use of this cut-point in our study allows for comparability with other accelerometer-based results from countries with accelerometry data of representative samples, that report prevalences based on this cut-point (e.g., the prevalence of activity based on this cut-point is higher in Mexico in comparison to the U.S., but lower than Canada) [11, 13, 29].

**Fig. 1:**
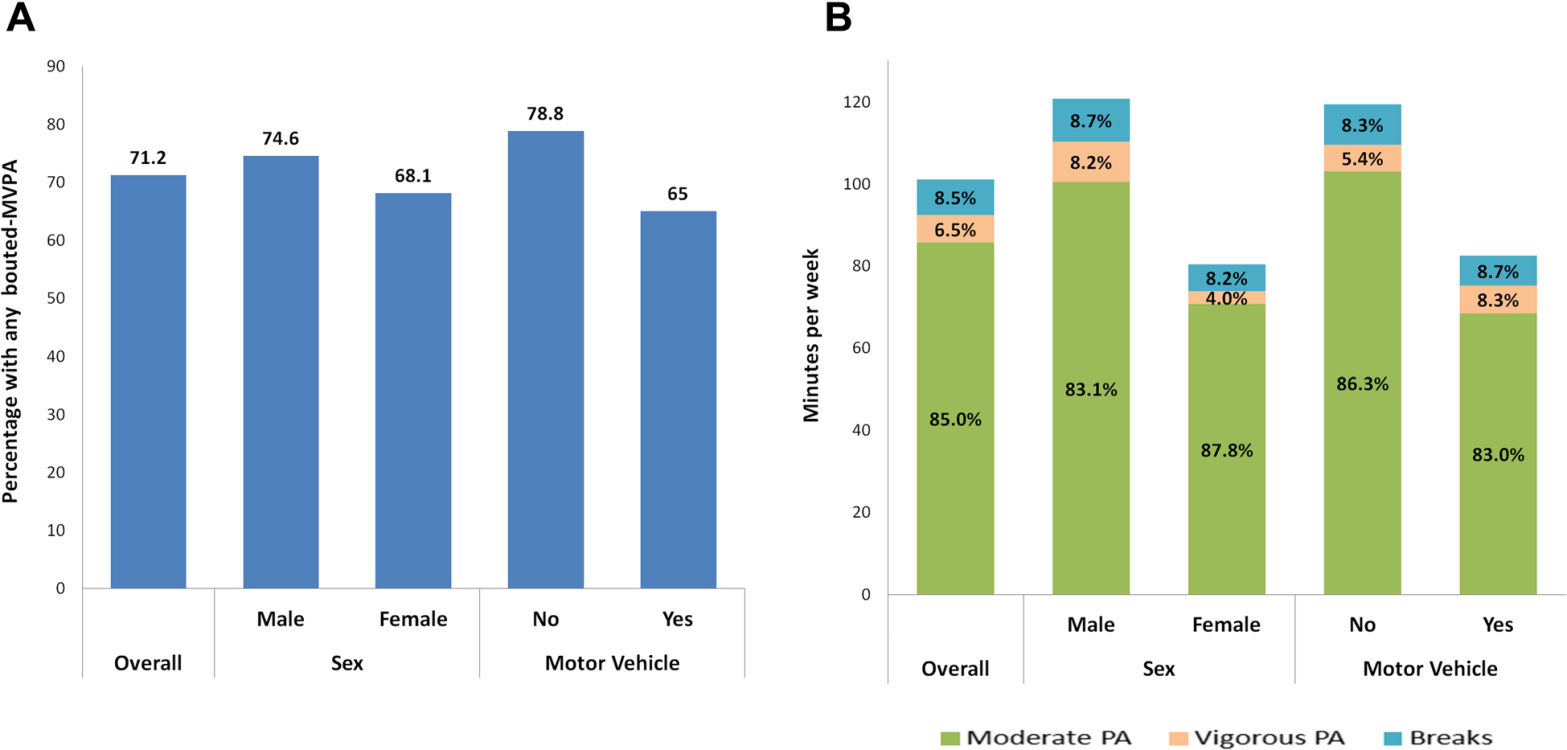
Prevalence, length and composition of MVPA bouts among Mexican adults from Cuernavaca, Mexico, 2011. **a**. Percentage of Mexican adults from Cuernavaca (2011) with any MVPA within bouts (i.e. ≥1 valid MVPA bout), by sex and motor vehicle ownership. **b**. Length and composition of MVPA bouts by sex and motor vehicle ownership. Bouts are defined as having a minimum duration of ten consecutive minutes, with 80 % of the bout corresponding to MVPA (Bouts A)

Since the importance of considering PA within bouts is well recognized and more studies on this topic are emerging, it is important to know the proportion of the population engaging in any bouted-PA at all, and how bouts are characterized in length and composition. Figure 1 (Additional file 1) examines this in more detail for BMVPA, stratifying by sex and motor vehicle ownership, the strongest predictors of BMVPA in our study. Fewer females and motor vehicle owners registered any BMVPA compared to men and non-motor vehicle owners, respectively. Furthermore, among adults with any BMVPA, females and motor vehicle owners had fewer minutes per week of BMVPA. Therefore, both were less likely to engage in any prolonged episode of MVPA (BMVPA), and among those who did, the duration of bouts of activity was lower than that of their counterparts (men and non-vehicle owners). The proportion of bout-time per week corresponding to breaks is stable across sexes and motor vehicle ownership status, varying from 8.2 to 8.7 %. Yet, a higher proportion of weekly bout-time is spent in BVPA among men compared to women (8.2 vs. 4.0 %), while among motor vehicle owners there is a higher percentage of BVPA in comparison to non-vehicle owners (8.3 vs. 5.4 %). The proportion of both TVPA and BVPA over TMVPA and TBMVPA, respectively, is very small among Mexican adults, and consistent with US data [11, 28, 30]. In contrast, MPA is considerably more widespread among the Mexican adult population, suggesting that public health efforts might better focus on promoting MPA rather than VPA, since MPA is achievable through brisk walking and is likely more feasible to increase at a population level through adequate promotion strategies [31].

Mexican adults are more active during weekdays versus weekends, suggesting that transport and occupational PA are larger contributors to MVPA than leisure-time PA among Mexican adults. This likely reflects that PA among Mexicans is driven by necessity rather than by choice [9]. This hypothesis should be further studied using domain-specific PA data available from this and other studies. Our results may help inform policy makers to target programs and interventions to increase PA during weekends and leisure time among Mexican adults.

The inverse relationship between PA and motor vehicle ownership is consistent with recent findings from several countries contrasting the levels of activity between private and public transport users. Car ownership has been negatively associated with activity levels and positively correlated with obesity [32–36]. A possible explanation for our findings may be that this is due to more transport-related PA (walking) taking place among the non-vehicle owners, which supports a need-based framework for understanding PA in Mexico (versus the more common choice-based framework) [9]. This hypothesis is also supported by the significant negative association of motor vehicle ownership only with BMPA (possibly representing walking) and not with BVPA. Meanwhile, the higher percentage of BVPA among vehicle owners (independent of SES) may imply that their BMVPA is more leisure than transport-related. Findings from Colombia suggest that access to public transportation is associated with both leisure and transport PA [37]. Our findings stress the need to promote leisure-time PA among Mexicans, and to creatively incentivize PA among motor vehicle owners through carefully thought multi-level strategies (e.g., economic incentives, adequate infrastructure for active transit, efficient, modern and safe public transit systems, etc.).

While the higher level of MVPA among men is consistent with findings from HIC [12, 38, 39], other results may be more context specific. SES was not associated with total or bout-specific MVPA for any of the intensity-specific PA outcomes in the adjusted analyses. Meanwhile, higher education (independent of SES) was associated with more VPA minutes per week, possibly reflecting more opportunities and awareness of the importance of leisure-time PA among highly educated Mexicans. There may be specific social constructs among Mexicans supporting this type of behavior, independent of wealth, but related to higher education levels. Another interesting finding was the null association of age with BMVPA after adjusting for all other covariates. Findings from HIC show an inverse relationship between age and MVPA [12, 39, 40]. In Mexico, a significant association was found for the highest age group only for TMPA, but not for BMPA, (Fig. 1). Therefore, among older Mexicans, the amount of non-bouted or sporadic MPA decreases in comparison to the younger group, yet no difference occurs for PA within bouts, which is most relevant for health maintenance. Further studies are needed to understand these relationships.

This study had several limitations. The cross sectional design did not allow determination of causality. Most socio-demographic variables (except BMI) were based on self-report, perhaps decreasing precision. The sample is only representative of adults from the city of Cuernavaca, and not for all Mexicans. Yet the similar rates of overweight and obesity to nationally representative data (72.6 % vs. 71.2 %) suggest comparability to the overall urban Mexican population [5]. We only addressed basic socio-demographic correlates of PA, but did not examine psychosocial and environmental correlates of PA. Further analyses using other levels of variables and their associations with objectively measured PA are needed for Mexico.

Our study had several strengths as well. This is the first study reporting objectively measured PA levels for a representative sample of Mexican adults, and for a Latin American country [8]. The data collection and scoring protocol was standardized with that of a multinational study (IPEN), using state of the art procedures [21, 25]. Our definition of bouts was consistent with recent approaches that consider a bout to be valid when 80 % of it corresponds to MVPA (allowing for each break to have a maximum duration of two minutes) [14], in contrast with the more traditional definition allowing for a maximum break time of two minutes throughout an entire bout of any duration [41, 42]. This approach enables the identification of more bouts of activity that may be occurring in real life situations (e.g. walking in an urban setting with occasional interruptions). Our study also provided further insight on the proportion of MPA and VPA within MVPA bouts. Finally, the use of weekly minutes of bouted and total PA as outcomes responded to the identified need for more studies treating PA as a continuous variable [12].

## Conclusions

A very low percentage of adults in Cuernavaca achieve at least 150 minutes per week of accelerometer-derived PA within bouts. These findings are consistent with those from HIC [11, 28, 29], highlighting the need for more health outcome studies in which PA is measured objectively, to generate standardized international recommendations of PA based on objective measures rather than applying a standard cut-point (150 min per week of MVPA) based on self-report. Meanwhile, subjective measures will remain a valuable means to complement objective tools by providing information on domain-specific PA that is useful for intervention and program design. Our study identified some contrasting results in comparison to those reported for HIC, highlighting the need for more high quality epidemiologic PA studies from LMIC. Up to now, our study provides the best available evidence on the levels of inactivity of Mexican adults from urban settings, and of the subgroups (women, motor-vehicle owners) to be targeted for PA promotion. Additional studies are needed to fully understand the intensity-specific relationships found in our analyses. A high proportion (81 to 94 %) of the variability of the studied PA outcomes remains unexplained. Future studies should more fully examine the psychosocial, economic, environmental and political determinants of PA among Mexicans.

## Additional file

Additional file 1:
**Means, quartiles and prevalences of intensity-specific, objectively measured physical activity by sex and age among adults from Cuernavaca, Mexico, 2011.**
