# Supplementary material for: Accelerometer-based physical activity levels among Mexican adults and their relation with sociodemographic characteristics and BMI: a cross-sectional study
Source: Int J Behav Nutr Phys Act. 2015 Jun 20;12:79. doi: 10.1186/s12966-015-0243-z (PMC4506632; doi:10.1186/s12966-015-0243-z)
Supplement: Additional file 1: — Means, quartiles and prevalences of intensity-specific, objectively measured physical activity by sex and age among adults from Cuernavaca, Mexico, 2011. [file 12966_2015_243_MOESM1_ESM.docx]

**Additional file 1: Means, quartiles and prevalences of intensity-specific, objectively measured physical activity by sex and age among adults from Cuernavaca, Mexico, 2011.**

| **Outcome** | ***Overall*** | | | | | ***<=35 years*** | | | | | ***35<years<=50*** | | | | | ***50<years<=65*** | | | | |
| --- | --- | --- | --- | --- | --- | --- | --- | --- | --- | --- | --- | --- | --- | --- | --- | --- | --- | --- | --- | --- |
|  | Mean/ % | SE / n | Q1 | Med | Q3 | Mean/ % | SE / n | Q1 | Med | Q3 | Mean/ % | SE / n | Q1 | Med | Q3 | Mean/ % | SE / n | Q1 | Med | Q3 |
| ***Overall*** |  |  |  |  |  |  |  |  |  |  |  |  |  |  |  |  |  |  |  |  |
| **Total Mins/Week of Activity** |  |  |  |  |  |  |  |  |  |  |  |  |  |  |  |  |  |  |  |  |
| Moderate | 214.7 | 9.7 | 97.0 | 176.2 | 296.9 | 238.1 | 14.1 | 125.0 | 207.4 | 320.1 | 215.3 | 11.6 | 103.4 | 185.9 | 301.5 | 185.9 | 14.8 | 60.5 | 131.0 | 234.0 |
| Vigorous | 6.6 | 0.8 | 0.0 | 0.0 | 2.2 | 10.9 | 2.2 | 0.0 | 0.9 | 8.1 | 4.7 | 1.0 | 0.0 | 0.0 | 2.2 | 4.2 | 1.4 | 0.0 | 0.0 | 0.0 |
| Moderate-to-Vigorous | 221.3 | 10.0 | 97.5 | 178.3 | 305.5 | 249.0 | 15.6 | 127.8 | 214.2 | 327.6 | 220.0 | 11.8 | 107.1 | 188.5 | 309.7 | 190.1 | 14.4 | 60.5 | 135.1 | 247.6 |
| **% with any MVPA in bouts^a^** | 71.2 | 469 | --- | --- | --- | 78.1 | 170 | --- | --- | --- | 72.4 | 184 | --- | --- | --- | 61.4 | 115 | --- | --- | --- |
| Number of bouts^a^ per week^b^ | 5.9 | 0.4 | 2.0 | 4.1 | 6.9 | 6.0 | 0.5 | 2.0 | 4.5 | 7.1 | 5.1 | 0.4 | 1.9 | 3.3 | 6.6 | 6.8 | 0.5 | 1.9 | 4.5 | 6.9 |
| Average bout^a^ duration^b^ | 17.0 | 0.5 | 12.3 | 14.7 | 18.2 | 16.3 | 0.8 | 12.1 | 14.5 | 17.8 | 17.0 | 0.8 | 12.0 | 14.6 | 18.2 | 18.0 | 0.8 | 12.4 | 15.0 | 20.3 |
| **% with no MVPA in bouts** | 28.8 | 200 | --- | --- | --- | 21.9 | 48 | --- | --- | --- | 27.6 | 75 | --- | --- | --- | 38.6 | 77 | --- | --- | --- |
| **Mins/Week of Activity-bouts^a^**  (Total Sample) | | | |  |  |  |  |  |  |  |  |  |  |  |  |  |  |  |  |  |
| Activity Breaks | 6.1 | 0.6 | 0.0 | 2.3 | 8.1 | 6.4 | 0.8 | 0.0 | 3.8 | 9.1 | 5.8 | 0.6 | 0.0 | 2.3 | 7.6 | 6.2 | 1.1 | 0.0 | 1.7 | 7.7 |
| Moderate Activity | 61.1 | 4.5 | 0.0 | 29.6 | 79.2 | 62.4 | 6.6 | 8.1 | 44.2 | 86.3 | 55.7 | 4.8 | 0.0 | 27.8 | 75.6 | 67.1 | 7.1 | 0.0 | 15.4 | 77.5 |
| Vigorous Activity | 4.7 | 0.6 | 0.0 | 0.0 | 0.0 | 7.1 | 1.5 | 0.0 | 0.0 | 2.5 | 3.4 | 0.9 | 0.0 | 0.0 | 0.0 | 3.7 | 1.4 | 0.0 | 0.0 | 0.0 |
| Moderate-to-Vigorous | 65.8 | 4.7 | 0.0 | 30.0 | 86.5 | 69.5 | 7.4 | 8.5 | 45.7 | 95.2 | 59.1 | 5.1 | 0.0 | 28.4 | 81.5 | 70.8 | 6.9 | 0.0 | 15.4 | 85.9 |
| **Mins/Week of Activity-bouts^a^**  (any MVPA in bouts=yes)^b^ | | | | | |  |  |  |  |  |  |  |  |  |  |  |  |  |  |  |
| Activity Breaks | 8.6 | 0.7 | 2.1 | 5.3 | 10.3 | 8.2 | 0.9 | 2.2 | 5.6 | 10.2 | 8.0 | 0.8 | 2.1 | 4.8 | 9.6 | 10.1 | 1.6 | 2.2 | 5.1 | 11.8 |
| Moderate Activity | 85.8 | 5.2 | 24.4 | 55.3 | 114.3 | 79.9 | 6.7 | 25.0 | 57.1 | 109.1 | 76.8 | 5.9 | 24.2 | 50.1 | 103.8 | 109.3 | 9.6 | 23.3 | 65.4 | 129.9 |
| Vigorous Activity | 6.6 | 0.9 | 0.0 | 0.0 | 2.2 | 9.0 | 2.0 | 0.0 | 0.0 | 5.8 | 4.7 | 1.2 | 0.0 | 0.0 | 1.9 | 6.1 | 2.3 | 0.0 | 0.0 | 0.0 |
| Moderate-to-Vigorous | 92.4 | 5.4 | 25.1 | 58.3 | 120.9 | 89.0 | 7.9 | 26.2 | 58.2 | 121.0 | 81.6 | 6.2 | 24.9 | 53.2 | 114.3 | 115.4 | 9.2 | 23.3 | 69.5 | 137.1 |
| ***Male*** |  |  |  |  |  |  |  |  |  |  |  |  |  |  |  |  |  |  |  |  |
| **Total Mins/Week of Activity** |  |  |  |  |  |  |  |  |  |  |  |  |  |  |  |  |  |  |  |  |
| Moderate Activity | 259.4 | 13.4 | 121.1 | 213.8 | 349.1 | 286.7 | 17.5 | 150.0 | 267.7 | 357.1 | 237.1 | 13.4 | 113.3 | 194.6 | 337.1 | 255.0 | 29.0 | 109.8 | 163.7 | 326.2 |
| Vigorous Activity | 10.7 | 1.4 | 0.0 | 0.9 | 8.0 | 18.5 | 3.7 | 0.0 | 2.0 | 17.1 | 5.1 | 1.4 | 0.0 | 0.0 | 4.0 | 8.5 | 3.1 | 0.0 | 0.0 | 2.8 |
| Moderate-to-Vigorous | 270.1 | 13.9 | 127.8 | 232.7 | 372.4 | 305.2 | 19.8 | 151.4 | 285.8 | 398.2 | 242.2 | 14.0 | 115.0 | 206.2 | 338.8 | 263.5 | 28.4 | 112.1 | 170.4 | 335.3 |
| **% with any MVPA in bouts^a^** | 74.6 | 226 | --- | --- | --- | 82.7 | 84 | --- | --- | --- | 70.5 | 81 | --- | --- | --- | 70.0 | 61 | --- | --- | --- |
| Number of bouts^a^ per week^b^ | 7.1 | 0.4 | 2.1 | 5.2 | 9.3 | 7.4 | 0.7 | 2.2 | 5.7 | 10.3 | 5.2 | 0.5 | 1.4 | 3.3 | 7.8 | 9.2 | 0.8 | 3.1 | 5.4 | 11.6 |
| Average bout^a^ duration^b^ | 16.8 | 0.7 | 12.8 | 14.9 | 17.8 | 16.9 | 0.4 | 12.8 | 14.6 | 17.8 | 16.2 | 0.6 | 13.3 | 14.7 | 17.7 | 17.6 | 1.1 | 13.4 | 15.0 | 18.0 |
| **% with no MVPA in bouts** | 25.2 | 76 | --- | --- | --- | 17.3 | 15 | --- | --- | --- | 29.5 | 35 | --- | --- | --- | 30.0 | 26 | --- | --- | --- |
| **Mins/Week of Activity-bouts^a^**  (Total Sample) | | | |  |  |  |  |  |  |  |  |  |  |  |  |  |  |  |  |  |
| Activity Breaks | 7.8 | 0.9 | 0.0 | 3.9 | 9.8 | 8.1 | 1.2 | 1.1 | 5.7 | 10.3 | 5.9 | 0.9 | 0.0 | 2.4 | 7.5 | 10.0 | 2.0 | 0.0 | 3.5 | 11.0 |
| Moderate Activity | 74.9 | 7.4 | 0.0 | 43.5 | 102.5 | 80.7 | 9.9 | 9.8 | 56.2 | 119.7 | 52.1 | 5.9 | 0.0 | 29.0 | 70.7 | 98.0 | 15.0 | 0.0 | 47.1 | 109.9 |
| Vigorous Activity | 7.4 | 1.1 | 0.0 | 0.0 | 2.2 | 11.8 | 2.5 | 0.0 | 0.0 | 8.0 | 3.0 | 1.1 | 0.0 | 0.0 | 1.1 | 7.6 | 3.1 | 0.0 | 0.0 | 1.4 |
| Moderate-to-Vigorous | 82.2 | 7.6 | 0.0 | 45.2 | 113.2 | 92.5 | 10.9 | 13.2 | 58.3 | 157.2 | 55.0 | 6.2 | 0.0 | 29.0 | 76.2 | 105.7 | 14.2 | 0.0 | 52.4 | 126.0 |
| **Mins/Week of Activity-bouts^a^**  (any MVPA in bouts=yes)^b^ | | | | | |  |  |  |  |  |  |  |  |  |  |  |  |  |  |  |
| Activity Breaks | 10.5 | 1.1 | 2.8 | 6.4 | 11.6 | 9.8 | 1.2 | 3.1 | 7.2 | 11.3 | 8.3 | 1.1 | 2.2 | 5.5 | 9.9 | 14.3 | 2.4 | 3.3 | 7.9 | 12.8 |
| Moderate Activity | 100.4 | 8.0 | 31.4 | 62.0 | 129.7 | 97.6 | 8.9 | 43.7 | 70.6 | 131.2 | 73.9 | 6.9 | 25.4 | 46.5 | 112.3 | 140.1 | 16.3 | 43.9 | 67.6 | 181.1 |
| Vigorous Activity | 9.9 | 1.5 | 0.0 | 0.0 | 5.9 | 14.3 | 3.4 | 0.0 | 1.5 | 13.9 | 4.2 | 1.6 | 0.0 | 0.0 | 2.0 | 10.9 | 4.5 | 0.0 | 0.0 | 3.8 |
| Moderate-to-Vigorous | 110.3 | 8.0 | 32.2 | 66.8 | 156.9 | 111.9 | 10.7 | 43.7 | 75.6 | 163.0 | 78.1 | 7.3 | 26.2 | 51.6 | 116.1 | 151.0 | 14.5 | 43.9 | 76.2 | 214.1 |
| ***Female*** |  |  |  |  |  |  |  |  |  |  |  |  |  |  |  |  |  |  |  |  |
| **Total Mins/Week of Activity** |  |  |  |  |  |  |  |  |  |  |  |  |  |  |  |  |  |  |  |  |
| Moderate Activity | 172.5 | 7.4 | 74.0 | 146.6 | 236.4 | 187.8 | 12.9 | 98.0 | 177.4 | 237.0 | 196.4 | 13.6 | 89.9 | 161.4 | 291.7 | 120.5 | 10.1 | 52.9 | 85.3 | 159.5 |
| Vigorous Activity | 2.8 | 0.5 | 0.0 | 0.0 | 1.0 | 3.0 | 0.6 | 0.0 | 0.0 | 1.1 | 4.3 | 1.3 | 0.0 | 0.0 | 1.3 | 0.2 | 0.1 | 0.0 | 0.0 | 0.0 |
| Moderate-to-Vigorous | 175.2 | 7.5 | 74.0 | 148.1 | 236.9 | 190.8 | 13.2 | 98.0 | 177.5 | 238.0 | 200.7 | 13.9 | 89.9 | 173.7 | 296.6 | 120.7 | 10.1 | 52.9 | 85.3 | 163.3 |
| **% with any MVPA in bouts^a^** | 68.1 | 243 | --- | --- | --- | 73.4 | 86 | --- | --- | --- | 74.1 | 103 | --- | --- | --- | 53.3 | 54 | --- | --- | --- |
| Number of bouts^a^ per week^b^ | 4.6 | 0.3 | 1.3 | 3.1 | 5.8 | 4.3 | 0.4 | 1.3 | 3.3 | 5.5 | 5.1 | 0.5 | 2.0 | 3.2 | 6.3 | 4.0 | 0.5 | 1.1 | 2.5 | 5.0 |
| Average bout^a^ duration^b^ | 17.1 | 0.7 | 11.8 | 14.5 | 19.3 | 15.5 | 0.4 | 11.6 | 14.5 | 17.7 | 17.6 | 1.2 | 11.9 | 14.4 | 20.7 | 18.6 | 1.2 | 10.9 | 14.9 | 23.2 |
| **% with no MVPA in bouts** | 33.8 | 124 | --- | --- | --- | 29.6 | 33 | --- | --- | --- | 25.9 | 40 | --- | --- | --- | 46.7 | 51 | --- | --- | --- |
| **Mins/Week of Activity-bouts^a^** (Total Sample) | | | |  |  |  |  |  |  |  |  |  |  |  |  |  |  |  |  |  |
| Activity Breaks | 4.5 | 0.4 | 0.0 | 2.0 | 6.1 | 4.5 | 0.7 | 0.0 | 2.3 | 5.8 | 5.7 | 0.7 | 0.0 | 2.3 | 7.9 | 2.6 | 0.3 | 0.0 | 0.0 | 2.3 |
| Moderate Activity | 48.1 | 4.9 | 0.0 | 23.0 | 65.6 | 43.5 | 5.9 | 0.0 | 25.3 | 61.6 | 58.8 | 7.3 | 0.0 | 27.4 | 78.6 | 37.9 | 6.9 | 0.0 | 6.0 | 43.5 |
| Vigorous Activity | 2.2 | 0.5 | 0.0 | 0.0 | 0.0 | 2.1 | 0.5 | 0.0 | 0.0 | 0.0 | 3.8 | 1.3 | 0.0 | 0.0 | 0.0 | 0.0 | 0.0 | 0.0 | 0.0 | 0.0 |
| Moderate-to-Vigorous | 50.3 | 5.1 | 0.0 | 23.1 | 72.6 | 45.6 | 6.1 | 0.0 | 25.9 | 61.6 | 62.6 | 8.0 | 0.0 | 27.6 | 82.8 | 37.9 | 6.9 | 0.0 | 6.0 | 43.5 |
| **Mins/Week of Activity-bouts^a^** (any MVPA in bouts=yes)^b^ | | | | | |  |  |  |  |  |  |  |  |  |  |  |  |  |  |  |
| Activity Breaks | 6.6 | 0.5 | 2.0 | 4.2 | 8.6 | 6.2 | 0.6 | 2.0 | 4.4 | 7.9 | 7.7 | 0.9 | 2.1 | 4.5 | 9.1 | 4.9 | 0.6 | 1.1 | 2.3 | 8.0 |
| Moderate Activity | 70.7 | 5.9 | 20.9 | 46.8 | 93.7 | 59.2 | 5.7 | 23.3 | 46.1 | 67.8 | 79.3 | 8.2 | 23.5 | 51.2 | 100.7 | 71.1 | 10.8 | 12.8 | 41.4 | 104.2 |
| Vigorous Activity | 3.2 | 0.7 | 0.0 | 0.0 | 0.0 | 2.9 | 0.6 | 0.0 | 0.0 | 0.0 | 5.1 | 1.7 | 0.0 | 0.0 | 1.1 | 0.1 | 0.1 | 0.0 | 0.0 | 0.0 |
| Moderate-to-Vigorous | 73.9 | 6.1 | 21.7 | 47.9 | 97.2 | 62.1 | 5.8 | 23.3 | 46.9 | 75.5 | 84.4 | 9.0 | 24.2 | 55.2 | 107.5 | 71.2 | 10.9 | 12.8 | 41.4 | 104.7 |

^a^ *Only activity registered within MVPA-bouts of at least 10 minutes duration, with ≥80% corresponding to MVPA (Bouts A) is reported. ^b^ Reported figures are only for participants with any MVPA within bouts (MVPA within bouts>0). Q1=Quartile 1, 25th percentile, Med=Median, 50th percentile, Q3=Quartile 3, 75th percentile. NOTE: All values are weighted for selection probability and non-response by sex*
